# Early Mode of Life and Hatchling Size in Cephalopod Molluscs: Influence on the Species Distributional Ranges

**DOI:** 10.1371/journal.pone.0165334

**Published:** 2016-11-09

**Authors:** Roger Villanueva, Erica A. G. Vidal, Fernando Á. Fernández-Álvarez, Jaruwat Nabhitabhata

**Affiliations:** 1 Institut de Ciències del Mar (CSIC), Passeig Marítim de la Barceloneta, Barcelona, Spain; 2 Centro de Estudos do Mar, Universidade Federal do Paraná (UFPR), Pontal do Paraná, Brazil; 3 Excellence Centre for Biodiversity of Peninsular Thailand (CBIPT), Faculty of Science, Prince of Songkla University, Hatyai, Songkhla, Thailand; Naturhistoriska riksmuseet, SWEDEN

## Abstract

Cephalopods (nautiluses, cuttlefishes, squids and octopuses) exhibit direct development and display two major developmental modes: planktonic and benthic. Planktonic hatchlings are small and go through some degree of morphological changes during the planktonic phase, which can last from days to months, with ocean currents enhancing their dispersal capacity. Benthic hatchlings are usually large, miniature-like adults and have comparatively reduced dispersal potential. We examined the relationship between early developmental mode, hatchling size and species latitudinal distribution range of 110 species hatched in the laboratory, which represent 13% of the total number of live cephalopod species described to date. Results showed that species with planktonic hatchlings reach broader distributional ranges in comparison with species with benthic hatchlings. In addition, squids and octopods follow an inverse relationship between hatchling size and species latitudinal distribution. In both groups, species with smaller hatchlings have broader latitudinal distribution ranges. Thus, squid and octopod species with larger hatchlings have latitudinal distributions of comparatively minor extension. This pattern also emerges when all species are grouped by genus (n = 41), but was not detected for cuttlefishes, a group composed mainly of species with large and benthic hatchlings. However, when hatchling size was compared to adult size, it was observed that the smaller the hatchlings, the broader the latitudinal distributional range of the species for cuttlefishes, squids and octopuses. This was also valid for all cephalopod species with benthic hatchlings pooled together. Hatchling size and associated developmental mode and dispersal potential seem to be main influential factors in determining the distributional range of cephalopods.

## Introduction

The early life of marine organisms is a decisive phase as it determines survival and recruitment success dictating many aspects of the species population dynamics. The varying synchrony between hatching of larvae and plankton production means that hatching in the right place and time will be decisive for first feeding success and thus, survival and growth. Dispersal of the hatchlings away from their parent source will have an imperative role in this process. In fact, as noted by R. Nathan [1]: “*dispersal is a fundamental biological process with important implications at multiple scales of organization*: *for the survival*, *growth and reproduction of individuals; for the composition*, *structure and dynamics of populations and communities; and for the persistence*, *evolution and geographical distribution of species*”. Multiple biotic and abiotic factors regulate dispersal and geographical distribution of marine species. Among them, the hatchling mode of life, benthic or planktonic, can be of notable influence on dispersal [2, 3]. However, few studies have addressed this topic, particularly for marine carnivores. Cephalopods play an important role as predators and prey in marine ecosystems [4] and their relative abundances have increased worldwide in recent years [5]. For adult cephalopods, both latitude and depth range have a significant effect on maximum body size, and temperature seems to be the most important factor in determining the distribution of adult body size along the continental shelves of the Atlantic Ocean [6]. These molluscs have both planktonic and benthic hatchling modes of life depending on the species. For this reason they may be used as models to improve our understanding of the factors that influence marine species distributional ranges. Most of the cephalopod species that have small hatchlings are planktonic during their early life, while those that produce larger hatchlings are usually benthic, with some exceptions.

Depending on the environment in which they live and their early mode of life, cephalopods can be divided into the following groups: holobenthic, when the full life cycle is associated with the benthos (e.g. most cuttlefishes); holopelagic, when the full life cycle is associated with the pelagic environment (e.g., all squids); and merobenthic, when hatchlings are planktonic followed by a benthic life from juvenile to adult (e.g., some octopuses). All three types of life cycle are observed in the octopods, a group with holopelagic, holobenthic and merobenthic representative species [7]. In comparison with other molluscs, cephalopods have direct development, thus hatchlings are essentially miniature of the adults and there are no marked morphological changes during ontogeny [4]. Nevertheless, the term paralarva is used on an ecological context to refer to planktonic hatchlings that have a different mode of life than the adults [8] and, the term juvenile is commonly used to refer individuals after the end of the planktonic phase, as well as for benthic hatchlings

Planktonic hatchlings are transported by currents and an inverse relationship should be expected between hatchling size and dispersal potential. In fact, transport by currents has been shown to be a powerful dispersal mechanism for planktonic squid [9–14] and octopus paralarvae [15, 16]. In addition to this passive transport, the swimming capacities recorded in the laboratory for planktonic cephalopod hatchlings are within the range or higher than those found for the larval fishes [17–22], which could enhance their dispersal potential. In contrast, mark and recapture experiments with hatchlings of large, benthic cuttlefish, showed limited shallow water dispersal, as the individuals remain in the same or adjacent bays to those in which they hatched [23]. Molecular studies revealed some low-scale geographic population structure in cuttlefish species [24], supporting the suspected low dispersal abilities of this group.

As a working hypothesis, we aim to evaluate if hatchling size and early mode of life, planktonic or benthic, are related to the extent of the species distribution. To understand the possible influence of the early mode of life on the geographic distribution of cephalopods, we studied three parameters for each species: the mean hatchling size, their mode of early life (planktonic or benthic) and the latitudinal distribution range of the species. We also selected species with known hatchling size to obtain additional information on the duration of the planktonic phase and on the relative hatchling size in relation to adult size. These parameters are subject to relative variability, both at spatial and temporal scales. However, they can offer interesting insights to understanding early life history strategy, species dispersal potential and biogeography in this group of marine invertebrate predators.

## Materials and Methods

### Hatchling and adult size

The hatchling size data were obtained through an extensive literature review, selecting only laboratory studies that examined: egg masses spawned in the laboratory from females properly identified at the species level, *in vitro* fertilization experiments and laboratory-hatched individuals from properly identified egg masses collected from the wild (see Table 1). These criteria were necessary to avoid possible taxonomic misidentifications, size variations and unknown age determination from wild-collected hatchlings. Hatchling size was measured as mantle length (ML; mm) of fresh individuals.

**Table 1:** Summary of data and literature references of hatchling mantle length (ML) and latitudinal distribution range for the 110 cephalopod species analyzed in the present study.

Group/Species	Hatchling mode of life	Hatchling ML (mm)	Maximum adult ML (mm)	SHSI (%)	Southern and northern latitudinal distribution	Latitudinal range (km)
**Sepioids**						
*Euprymna berryi*	P	2.6 [48]	50	5.3	20°51'N, 41°38'N [49]	2311
*Euprymna hyllebergi*	P	2.2 [50]	35	6.3	00°17'N, 16°41'N [51]	1824
*Euprymna scolopes*	P	1.8 [52]	30	5.8	18°47'N, 25°47'N [53]	779
*Euprymna tasmanica*	B	5.0 [54]	40	12.5	43°42'S, 24°40'S [49]	2116
*Idiosepius biserialis*	P	1.0 [55] (as *I*.*thailandicus*)	10.5	9.5	26°00'S, 33°25'N [56]	6607
*Idiosepius paradoxus*	P	1.2 [57]	16	7.4	21°51'N, 42°87'N [56, 58]	2478
*Idiosepius pygmaeus*	P	1.0 [59]	20	5.0	25°11'S, 13°32'N [60, 61]	4306
*Metasepia pffeferi*	B	4.0 [62]	60	6.7	04°04'S, 32°33'S [63]	3166
*Metasepia tullbergi*	B	4.7 [64]	70	6.7	00°01'S, 38°58'N [63]	4335
*Rossia macrosoma*	B	5.5 [65]	85	6.5	12°36'N, 70°03'N [49]	6387
*Rossia mollicella*	B	7.8* [66]	36	21.7	30°57'N, 45°37'N [67]	1630
*Rossia pacifica*	B	6.0 [68]	90	6.7	28°00'N, 65°57'N [49]	4219
*Sepia apama*	B	12.1 [69]	500	2.4	22°55'S, 39°27'S [63]	1839
*Sepia bandensis*	B	8.0 [70]	70	11.4	08°48'S, 15°18'N [63]	2681
*Sepia esculenta*	B	5.2 [48, 71]	180	2.9	06°48'N, 40°16'N [63]	3723
*Sepia latimanus*	B	14.1 [72–74]	500	2.8	25°04'S, 40°22'N [63]	7325
*Sepia lycidas*	B	8.7 [48] (as *S*. *subaculeata*)	380	2.3	07°50'S, 40°23'N [63]	5363
*Sepia officinalis*	B	6.3 [75, 76]	490	1.3	15°04'N, 61°28'N [63]	5160
*Sepia orbignyana*	B	6.0 [77]	120	5.0	17°49'S, 54°21'N [63]	8024
*Sepiadarium kochii*	B	1.5 [78]	30	5.0	25°25'S, 36°05'N [79]	6840
*Sepiella inermis*	P	4.3 [80]	125	3.4	19°43'S, 29°30'N [63]	5474
*Sepiella japonica*	P	4.3 [48] (as *S*. *maindroni*), [81]	180	2.4	21°31'N, 40°18'N [63]	2087
*Sepietta neglecta*	B	2.5 [82]	33	7.6	25°16'N, 62°07'N [49]	4099
*Sepietta obscura*	B	2.2 [82]	30	7.3	32°00'N, 45°31'N [49]	1501
*Sepietta oweniana*	B	4.0 [28]	50	8.0	14°37'N, 71°15'N [49]	6296
*Sepiola affinis*	B	3.0 [83]	25	12.0	35°46'N, 45°33'N [49]	1089
*Sepiola atlantica*	P	1.7 [84, 85]	21	8.1	26°15'N, 65°50'N [49]	4401
*Sepiola ligulata*	B	2.3 [82]	25	9.2	35°17'N, 45°33'N [49]	1142
*Sepiola robusta*	B	2.2 [82, 86]	28	7.9	30°38'N, 45°39'N [49]	1668
*Sepiola rondeleti*	B	3.7 [82]	60	6.2	12°39'N, 62°21'N [49]	5527
**Squids**						
**Neritic squids**						
*Doryteuthis gahi*	P	3.0 [87, 88]	400	0.8	57°04'S, 03°48'S [26]	5923
*Doryteuthis opalescens*	P	2.7 [89–91]	305	0.9	22°42'N, 57°48'N [26]	3902
*Doryteuthis pealeii*	P	1.7 [20, 89, 92]	465	0.4	08°47'N, 46°47'N [26]	4226
*Doryteuthis plei*	P	1.5 [89]	370	0.4	35°00'S, 36°46'N [26]	7979
*Heteroligo bleekeri*	P	3.3 [71, 93, 94]	380	0.9	25°26'N, 45°41'N [26]	2252
*Loligo forbesii*	P	4.2 [95, 96]	937	0.4	19°58'N, 62°15'N [26]	4701
*Loligo reynaudii*	P	2.4 [97–100]	400	0.6	36°58'S, 28°00'S [26, 101]	997
*Loligo vulgaris*	P	3.3 [102–105]	640	0.5	19°10'S, 61°45'N [26]	8998
*Lolligunculla diomedeae*	P	1.3 [106]	115	1.1	31°37'N, 18°31'S [26, 107]	5573
*Loliolus japonica*	P	2.3 [103]	150	1.5	06°02'N, 40°25'N [26]	3824
*Loliolus sumatrensis*	P	1.5 [78]	120	1.3	09°08'S, 40°28'N [26]	5515
*Uroteuthis duvaucelii*	P	1.1 [78]	330	0.3	25°51'S, 28°58'N [26]	6095
*Uroteuthis edulis*	P	2.0 [71]	502	0.4	25°48'S, 53°33'N [26]	8823
*Sepioteuthis australis*	P	6.8* [108, 109]	394	1.7	43°46'S, 20°13'S [26]	2618
*Sepioteuthis sepioidea*	P	5.0 [110]	200	2.5	13°08'S, 28°21'N [26]	4613
**Oceanic squids**						
*Dosidicus gigas*	P	1.1 [111, 112]	1200	0.1	55°40'S, 58°08'N [42]	12650
*Gonatus madokai*	P	7.2* [113]	470	1.5	59°58'N, 40°35'N [114]	2156
*Gonatus onyx*	P	3.4 [115]	150	2.3	30°01'N, 60°00'N [116]	3335
*Illex argentinus*	P	1.6 [117]	400	0.4	55°24'S, 21°24'S [42]	3781
*Illex coindetii*	P	1.4 [118]	379	0.4	19°26'S, 61°18'N [42]	8978
*Illex illecebrosus*	P	1.2 [119, 120]	340	0.3	25°06'N, 67°14'N [42]	4686
*Ommastrephes bartramii*	P	1.3 [121]	600	0.2	20°00'N, 55°00'N [42]	3892
*Thysanoteuthis rhombus*	P	1.4 [122]	1000	0.1	43°51'S, 51°45'N [123]	10630
*Todarodes pacficus*	P	1.3 [124]	500	0.3	20°27'N, 62°29'N [42]	4674
*Todarodes sagittatus*	P	1.8 [125]	750	0.2	10°45'N, 72°20'N [42]	6848
*Todaropsis eblanae*	P	2.2 [125]	290	0.8	43°59'S, 73°34’N [42]	13070
*Watasenia scintillans*	P	1.4 [126]	70	2.0	22°54'N, 50°48'N [127]	3103
**Octopods**						
*Bolitaena pygmaea*	P	2.5* [128]	60	4.2	42°32’S, 37°12’N [129]	8866
*Vitreledonella richardi*	P	2.8* [130]	110	2.6	31°24’S, 48°55'N [129]	8930
*Tremoctopus violaceus*	P	1.9* [130]	250	0.8	36°01'S, 45°37'N [131]	9076
*Argonauta argo*	P	1.0* [130]	97	1.1	41°06'S, 45°28'N [132, 133]	9625
*Argonauta hians*	P	0.6 [134]	40	1.5	17°27'S, 45°36'N [133]	7012
*Amphioctopus aegina*	P	2.9 [135, 136]	90	3.2	03°03'S, 26°21'N [137]	3270
*Amphioctopus burryi*	P	1.5 [138]	70	2.2	13°00'S, 34°00'N [139, 140]	5227
*Amphioctopus fangsiao*	B	2.9 [141–143]	80	3.6	22°12'N, 42°33'N [137]	2262
*Amphioctopus neglectus*	P	2.8 [144]	64	4.4	01°19'N, 25°52'N [137]	2730
*Amphioctopus rex*	P	2.3 [145]	76	3.0	13°40'S,16°52'N [137, 146]	3394
*Bathypolypus bairdii*	B	7.7 [147]	70	11.0	27°01'N, 74°27'N [148]	5275
*Callistoctopus macropus*	P	4.0 [29]	155	2.6	16°02'S, 45°28'N [149]	6838
*Callistoctopus ornatus*	P	2.7* [150]	130	2.1	34°06'S, 34°49'N [151]	7662
*Cistopus indicus*	P	2.6 [144]	180	1.4	02°15'N, 07°44'N [137]	609
*Eledone cirrhosa*	P	4.5 [152]	250	1.8	30°32'N, 68°10′N [153]	4184
*Eledone moschata*	B	10.5 [30, 154]	140	7.5	30°32'N, 45°28'N [154]	1660
*Enteroctopus dofleini*	P	5.4 [155]	600	0.9	32°32'N, 62°31'N [156, 157]	3335
*Enteroctopus megalocyathus*	P	8.4 [158]	190	4.4	56°10'S, 34°20’S [158]	2428
*Graneledone boreopacifica*	B	28.0 [31]	145	19.3	40°28'N, 50°00'N [31, 159]	1061
*Hapalochlaena lunulata*	P	2.3 [160]	50	4.6	16°12'S, 18°28'N [137]	3854
*Hapalochlaena maculosa*	B	4.0 [161]	57	7.0	41°09'S, 38°06'S [60, 161]	339
*Octopus maorum*	P	4.6 [162, 163]	300	1.5	52°25'S, 31°40'S [163]	2307
*Octopus berrima*	B	4.5 [163]	105	4.3	43°41'S, 32°18'S [163]	1266
*Octopus bimaculoides*	B	6.5 [164]	85	7.6	22°32'N, 35°31'N [137]	1442
*Octopus bimaculatus*	P	2.6 [165]	200	1.3	22°51'N, 34°30'N [137]	1295
*Octopus briareus*	B	6.3 [164, 166]	120	5.2	02°49'S, 27°28'N [167]	3368
*Octopus chierchiae*	B	3.5 [168]	25	14.0	08°58'N, 29°33'N [169]	2288
*Octopus cyanea*	P	2.1* [150]	160	1.3	24°26'S, 22°15'N [170]	5192
*Octopus fitchi*	P	2.5* [130]	45	5.6	24°00'N, 32°00'N [171]	890
*Octopus hubbsorum*	P	1.2 [172]	220	0.6	04°00'N, 31°51'N [173]	3097
*Octopus huttoni*	P	2.5 [130, 174] (as *Robsonella australis*),[175]	57	4.3	50°45'S, 31°31'S [132]	2140
*Octopus insularis*	P	1.7 [176]	120	1.4	25°32ʹS, 00°56’N [177, 178]	2945
*Octopus joubini*	P	2.5 [179]	45	5.6	18°13'N, 30°03'N [179]	1317
*Octopus* sp. *"joubini"* undescribed	B	5.5 [180] (as *O*.*joubini*)	40	13.8	10°05'N, 30°03'N [179]	2221
*Octopus maya*	B	7.0 [164]	120	5.8	17°59'N, 21°32'N [137]	1268
*Octopus mimus*	P	1.9 [181]	190	1.0	33°54'S, 03°27'S [137]	3387
*Octopus minor*	B	10.0 [182]	80	12.5	21°57'N, 46°42'N [137, 183]	2752
*Octopus oliveri*	P	1.3* [184]	70	1.8	29°16'S, 27°06'N [185]	6267
*Octopus pallidus*	B	6.5 [163, 186]	150	4.3	44°00'S, 31°40'S [163]	1372
*Octopus rubescens*	P	1.9 [130]	100	1.9	22°51'N, 61°06'N [156]	4255
*Octopus salutii*	P	3.8 [187]	165	2.3	30°21'N, 50°00'N [137, 188]	2185
*Octopus tehuelchus*	B	6.6 [189]	200	3.3	44°01'S, 20°00'S [137, 190]	2670
*Octopus tetricus*	P	1.5 [191]	140	1.1	40°00'S, 28°12'S [137, 192]	1313
*Octopus superciliosus*	B	4.5 [163]	26	17.3	47°11'S, 33°14'S [163]	1552
*Octopus vulgaris sensu stricto*	P	2.1 [193, 194]	250	0.8	07°04'N, 53°03'N [137]	5113
*Octopus vulgaris* type II	P	2.2 [195]	210	1.1	31°05'S, 02°00’N [137]	3679
*Octopus vulgaris* type IV	P	2.1 [196]	168	1.3	21°28'N, 45°20'N [137]	2654
*Octopus warringa*	P	2.5 [163]	35	7.1	47°20'S, 33°33'S [163]	1531
*Octopus conispadiceus*	B	12.0 [197]	210	5.7	41°43'N, 45°37'N [198]	435
*Paroctopus digueti*	B	5.3 [164, 199]	74	7.2	22°48'N, 31°27'N [200]	963
*Robsonella fontanianus*	P	2.9 [201–203]	70	4.1	55°00'S, 06°00'S [204]	5449
*Scaeurgus unicirrhus*	P	2.0 [32]	90	2.2	26°10’S, 45°28'N [149, 205]	7965
*Wunderpus photogenicus*	P	2.3 [206]	36	6.4	16°15’S, 14°34'N [207]	3428

Hatchling mode of life as benthic (B) or planktonic (P) are indicated.

Hatchling mantle length (ML) are from fresh individuals, except for 10 species indicated by an asterisk (*), showing the estimated fresh ML from fixed individuals (see Methods for details). When the hatchling ML of a species was obtained from more than one study, the mean of each study was pooled to obtain the mean value representative of that particular species. Maximum adult ML, species hatchling size index (SHSI), the southern and northern latitudinal distribution as well as the latitudinal distribution range are also indicated for each species.

For each species and publication source, the hatchling ML (mm) was obtained as the mean value provided by the study. When the hatchling ML of a species was obtained from more than one study, the mean of each study was pooled to obtain the mean value representative of that particular species. The hatchling ML of a genus was obtained as the mean ML of all the species from that genus, following the FAO taxonomic criteria [25–27]. Hatchling ML from fresh individuals was selected when possible. When only preserved material existed for a species (n = 10 species, see Table 1), a shrinkage correction factor of 25.8% was applied to estimate the fresh ML. This correction factor was obtained as the average shrinkage percentage from five species whose fresh and preserved hatchling ML was known from the same individuals. These species were: *Sepietta oweniana* [28], *Callistoctopus macropus* [29], *Eledone moschata* [30], *Graneledone boreopacifica* [31] and *Scaeurgus unicirrhus* [32], with respective shrinkages of 37.5, 25.0, 10.0, 34.6 and 22.0%. Thus, when only measurements from preserved material was available, 25.8% of the preserved ML was added to estimate the fresh ML.

Some species with well-known hatchling size were excluded from the analysis for different reasons. This applied to species currently considered as a species complex, consequently, having uncertain taxonomic and latitudinal distribution such as *Sepia pharaonis* [33, 34], *Sepioteuthis lessoniana* [35–37], *Loligunculla brevis* [38] and *Sthenoteuthis oualaniensis* [39], as well as species with uncertain taxonomic status like *Pinnoctopus cordiformis* [40]. *Ommastrephes bartramii* is thought to represent at least three different species [41]. However, the hatchlings previously described in the literature are from one of this species, with a known distribution range in the North Pacific [42]. Hatchlings from species recently described and that have not been recorded outside the type locality were also excluded from the analysis, such as *Octopus laqueus* [43]. The particular characteristics and anatomy of the nautiluses, which possess very large hatchlings of 26–30 mm [44, 45], are measured through the shell diameter and not by with standard ML, as in other cephalopods. Thus, they were also excluded from the general comparative analysis.

The duration of the planktonic phase (in days, d) in cephalopod species obtained from culture experiments was recorded from the literature (Table 2). For comparisons between species, the maximum duration of the planktonic phase was selected for each species. The hatchling size in relation to the adult size was also explored as an indicator of the relative hatchling size for each species. Here we defined the species hatchling size index (SHSI, %) as: [(mean hatchling ML of the species)/(maximum adult ML of the species)]x100. The maximum adult ML was selected instead of the mean adult ML due to the high intraspecific variability of the latter in the literature. The maximum adult ML (in mm) was recorded in most cases based on recent FAO reviews [25–27], except for *Octopus vulgaris* type II [46] and type IV [47] (Table 1).

**Table 2:** Size at hatching and at the end of the planktonic phase, rearing temperature and duration of planktonic phase for 15 cephalopod species cultured in the laboratory.

Group/Species	Size at hatching (ML, mm)	Size at the end of planktonic phase (ML, mm)	Temperature (°C)	Duration of planktonic phase (d)
**Sepioids**				
*Sepiella inermis*	4.3	6.5	28	5 [80]
*Sepiola atlantica*	1.9	2.4	14.4 ± 0.3	6 [84]
*Euprymna hyllebergi*	2.2	Nd	28.2 ± 1.6	0.3 [50]
*Euprymna scolopes*	1.6–1.9	Nd	21–25	20–30 [52]
**Squids**				
*Doryteuthis opalescens*	2.3–2.8	15.0	15	60–80 [90]
* *	2.5–2.7	6.0–8.0	16±1	35–45 [208]
*Doryteuthis pealeii*	1.8	4.0–6.0	13–19	50–60 [92]
*Loligo forbesii*	3.4–4.9	5.3–9.0	12–15	40–50 [96]
*Sepioteuthis lessoniana*	5–6	12.0–30.0	24.5–25.5	30–60 [209]
	5.4	10.8	28	10 [210]
**Octopods**				
*Amphioctopus aegina*	2.7	6.3	29.7±0.6	20–25 [136]
*Enteroctopus dofleini*	5.3–5.5	13.5	10.8	100–117 [155]
	Nd	Nd	Nd	88 [211]
	Nd	Nd	11–11.5	150–180 [212, 213]
*Enteroctopus megalocyathus*	8.4 [158]	Nd	12	90–114 [214]
*Octopus joubini*	2.5	3.0–4.0	24	21 [179]
*Octopus vulgaris* sensu stricto	2.0	8.6	21.2	47–54 [193]
	Nd	Nd	22.5	40 [215]
	2.2	6.5	21.2	52–60 [194]
*Octopus vulgaris* type IV	2.1	6.3	24.7	33 [196]
*Robsonella fontanianus*	2.2	5.7	11	72 [202]

For squids, the end of the planktonic phase was considered to occur when schooling behaviour was first observed. ML, mantle length; Nd, no data; d, days.

The number of species used in the present study (n = 110) represents 13% of the 845 living cephalopod species described to date [216] and can be considered as a representative sample for this group of molluscs. The number of genus (n = 41) and families (n = 14) analysed here represents 24% and 28% respectively of the 174 genus and 50 families described to date for cephalopods [216]. Nevertheless, some groups may be over-represented in this sample, for example, the number of octopod species analysed here (n = 53) represents nearly half of the total number of species considered in the present study (n = 110), while the total number of octopod species represents nearly one-third of cephalopod species. This fact illustrates that this group is relatively easy to maintain and reproduce in aquaria, making it easier to collect egg masses and hatchlings in comparison with squids, which are more difficult to rear, spawn and consequently, to obtain hatchlings under laboratory conditions [217–219].

### Latitudinal distribution

Systematics and geographical distribution of the species were determined at first instance according to recent FAO reviews [25–27]. Then, for each species, an extensive literature review on its distributional range was conducted. The literature references used to obtain latitudinal distribution assigned to each species are found in Table 1. The maximum and minimum latitudinal distribution ranges obtained for each species was introduced in Google Earth^®^ to determine the latitudinal range of the species in degrees of latitude [220]. This range was transformed into distance (d) in km by applying the haversine formula to calculate the great-circle distance between two points; that is, the shortest distance over the earth’s surface. The haversine formula was used was: d = R·c; where, R = the earth’s radius (mean radius = 6371 km); c = 2·atan2(√a, √(1−a)); a = sin^2^(Δφ/2) + cos(φ_1_)·cos(φ_2_)·sin^2^(Δλ/2); φ, latitude; λ, longitude.

### Data treatment

Values were compared using analysis of variance (ANOVA) and differences were considered significant when P < 0.05. Linear regressions were used for the graphics. Data were assessed using the JMP statistical package.

## Results

**Fig 1:**
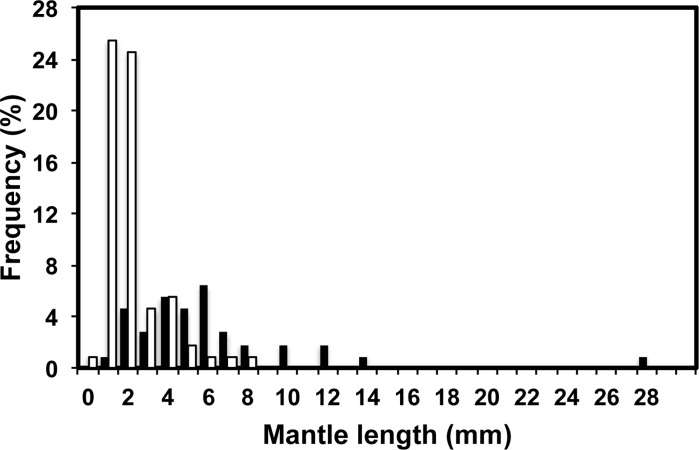
Size-frequency distribution from hatchlings of 110 species of cephalopods hatched in the laboratory. Empty columns represent planktonic hatchlings; black columns, benthic hatchlings.

Hatchling size was recorded for 110 species of cephalopods (30 sepioids, 27 squids, 53 octopods), ranging from 0.6 (*Argonauta hians*) to 28.0 (*Graneledone boreopacifica*) mm ML, with an average size of 3.9±3.5 mm ML. Species from 14 families and 41 genera are listed (Table 1). Of these, 38 species have benthic hatchlings and 72 species have planktonic phase of variable duration (Table 2). Sizes and ranges of planktonic and benthic hatchlings are shown in Table 3. The distribution of sizes for species with planktonic hatchlings showed a maximum for *Enteroctopus megalocyathus* (8.4 mm ML) and a minimum for *A*. *hians*. For species with benthic hatchlings, the smallest size was found in *Sepiadarium kochii* (1.5 mm ML) and the distribution of sizes was highly right-skewed due to the *G*. *boreopacifica* hatchling (Fig 1). There was a considerable overlap between the hatchling sizes of benthic and planktonic species for intermediate sizes classes, although the frequency of planktonic hatchlings in these classes was very low (Fig 1). Maximum adult size of the species analysed ranged from 10.5 (*Idiosepius biserialis*) to 1200 mm (*Dosidicus gigas*) ML, with an average size of 201±217 mm ML. The species hatchling size index (SHSI) ranged from 0.1 (*Thysanoteuthis rhombus*) to 21.7% (*Rossia molicella*), with an average of 4.3±4.2% (Table 1).

**Table 3:** Hatchling size in mantle length (ML) and latitudinal distribution ranges of 110 cephalopod species according to their hatchling mode of life as planktonic or benthic.

Hatchling mode	Hatchling ML (mm)			Latitudinal distribution (km)		
	mean±SD	min	max	mean±SD	min	max
Planktonic (n = 72)	2.5±1.5^a^	0.6	8.4	4684±2820^a^	609	13070
Benthic (n = 38)	6.5±4.6^b^	1.5	28.0	3105±2098^b^	339	8024

Values are mean±SD and different superscript letters denote statistical differences between planktonic and benthic (P<0.05).

In relation to the latitudinal distribution ranges, *Hapalochlaena maculosa* registered the smallest range (339 km) for a benthic species and the oceanic ommastrephid squid *Todaropsis eblanae* reached the broadest range for the planktonic species (13070 km) (Table 1). Species with a planktonic phase have significantly smaller hatchling sizes than species that hatched as benthic individuals. In addition, species with planktonic hatchlings display significantly broader latitudinal distribution compared with species that have benthic hatchlings (Table 3).

**Fig 2:**
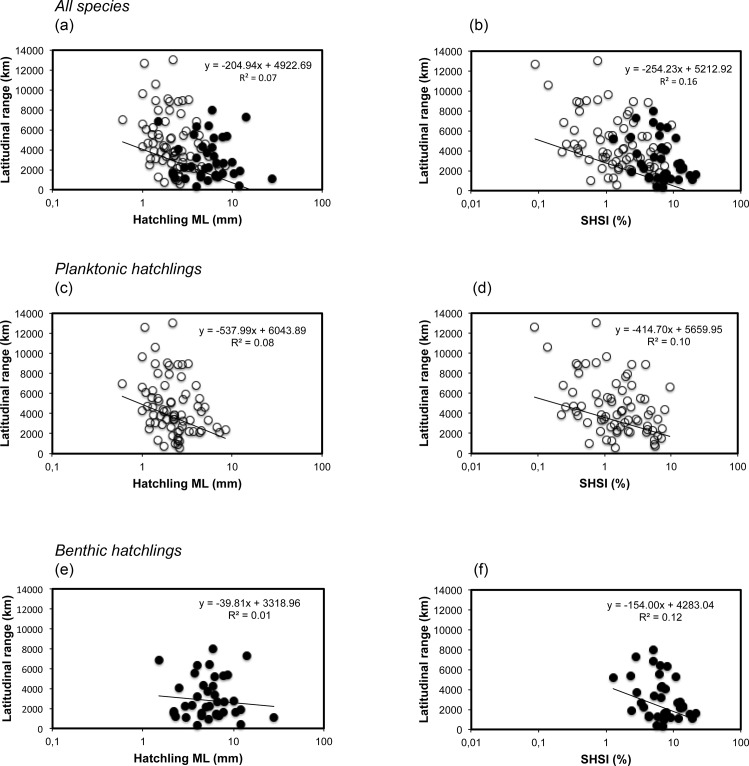
Relationship between hatchling size in mantle length (ML) and species hatchling size index (SHSI) with the latitudinal distribution range of cephalopod species hatched in the laboratory. a), c) and e) shows relationship between hatchling size and latitudinal distribution range of the species; b), d) and f), relationship between the SHSI and the latitudinal distribution range of the species. Data from a) and b) are based on all the 110 cephalopod species analysed in this study; data from c) and d) are based on 72 cephalopod species with planktonic hatchlings; data from e) and f) are based on 38 cephalopod species with benthic hatchlings. Logarithmic scale is used for the X-axis. Empty circles represent planktonic species; black circles benthic species.

**Fig 3:**
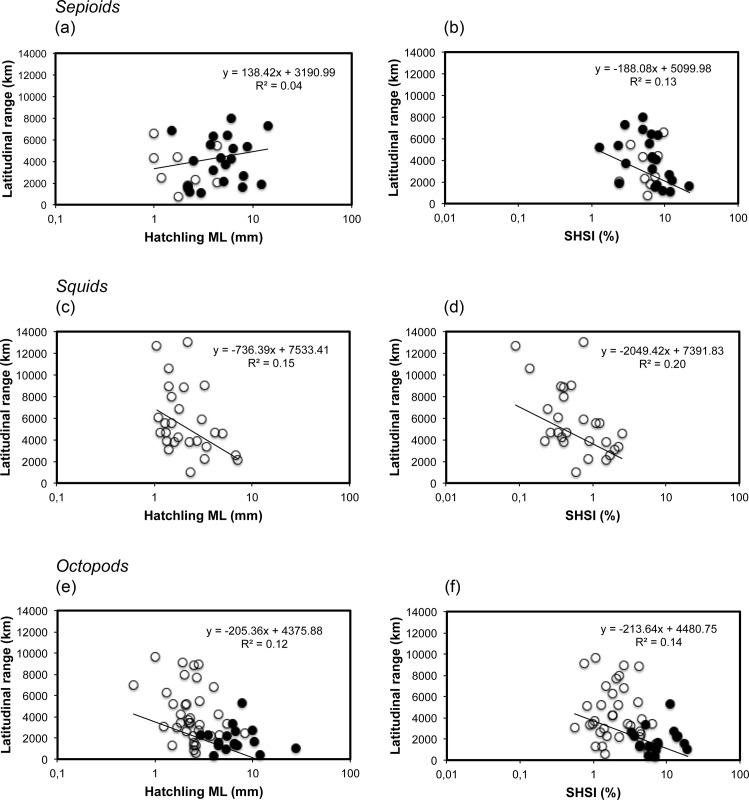
Relationship between hatchling size in mantle length (ML) and species hatchling size index (SHSI) with the latitudinal distribution range of cephalopod species hatched in the laboratory for the major cephalopod groups. a), c), e), relationship between hatchling size and latitudinal distribution range of the species; b), d), f), relationship between the SHSI and the latitudinal distribution range of the species. Data from a) and b) are based on 30 species of sepioids, data from c) and d) are based on 27 species of squids, and data from e) and f) are based on 53 species of octopods. Logarithmic scale is used for the X-axis. Empty circles represent planktonic species; black circles benthic species.

When all species were pooled together, a significant inverse relationship was observed between hatchling size and latitudinal distributional range, with species with large hatchlings showing smaller latitudinal distribution ranges (ANOVA, F = 7.17, p = 0.009, n = 110) (Fig 2A). The same relationship is observed when grouping all species at the genus level (F = 5.86, p = 0.02, n = 41). This inverse relationship was also significant when analysing all the planktonic species together, where species with smaller planktonic hatchlings reach broader distributions (F = 5.94, p = 0.017, n = 72) (Fig 2C). In contrast, no relationship between hatchling size and latitudinal distribution was found when all the benthic species were analysed together (F = 0.10, p = 0.76, n = 38) (Fig 2E). When the data were analysed between different major cephalopod groups, this relationship was not found for the major benthic cephalopod group, the sepioids (F = 2.61, p = 0.12, n = 30) (Fig 3A). For squids, a group with only planktonic hatchlings, an inverse relationship exists between hatchling size and latitudinal distribution, the smaller the paralarval size the broader the distributional range of the species (F = 4.36, p = 0.047, n = 27) (Fig 3C). For octopods, a cephalopod group with both planktonic and benthic hatchlings, an inverse relationship between hatchling size and species distributional range was also observed (F = 6.90, p = 0.01, n = 53) (Fig 3E).

When the SHSI is plotted against the latitudinal distribution range of species, the inverse relationship obtained was stronger than when using the mean hatchling size, showing a significant inverse relationship between the relative hatchling size and the latitudinal distribution range for all species (ANOVA, F = 21.45, p = 0.0001, n = 110) (Fig 2B) and for all genera (F = 8.09, p = 0.007, n = 41) pooled together. The same inverse relationship was also observed for all planktonic (F = 8.11, p = 0.006, n = 72) and all benthic (F = 6.51, p = 0.015, n = 38) species (Fig 2D and 2F). In relation to sepioids (F = 6.09, p = 0.02, n = 30), squids (F = 6.16, p = 0.02, n = 27) and octopods (F = 7.88, p = 0.007, n = 53), the same inverse relationship was found (Fig 3B, 3D and 3F).

**Fig 4:**
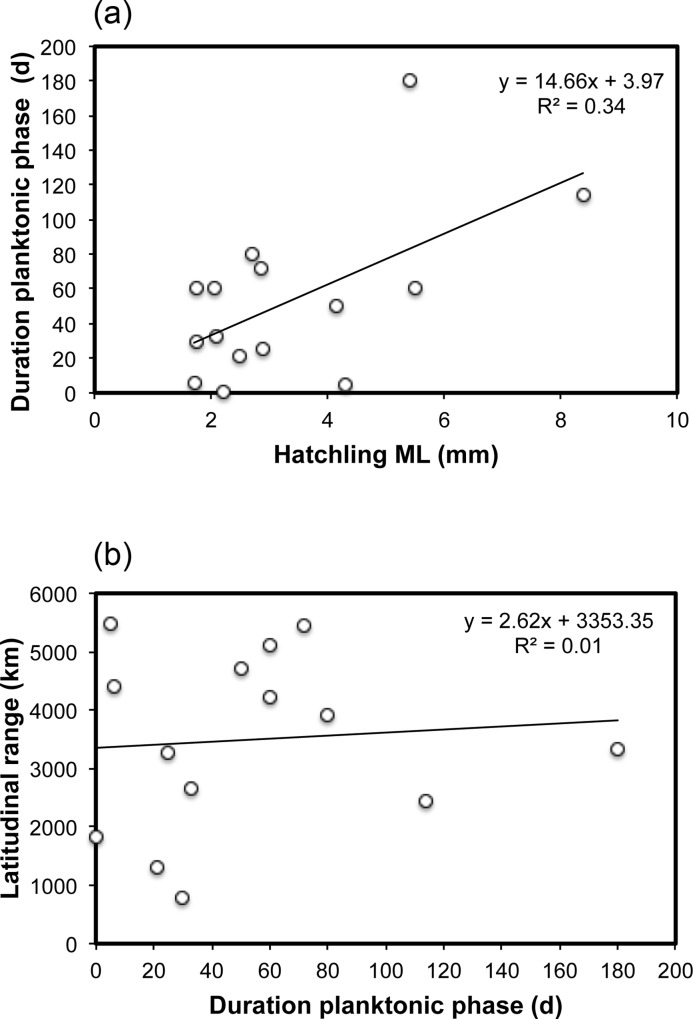
Relationships between (a) hatchling size in mantle length (ML) and duration of the planktonic phase for 15 cephalopod species; and (b) duration of planktonic phase versus latitudinal distribution range for 14 cephalopod species. See Table 2 for details.

The duration of the planktonic phase extended from less than a day (8h for *Euprymna hyllebergi*) to 180 d in *Enteroctopus dofleini*, with a mean of 53±47 d (Table 2). This duration increased with hatchling size (ANOVA, F = 6.74, p = 0.02, n = 15) (Fig 4A) and no relationship was found between duration of the planktonic phase and latitudinal range (F = 0.08, p = 0.78, n = 14) (Fig 4B). *Sepioteuthis lessoniana* was excluded from the latter comparison because it is considered a species complex [35–37].

## Discussion

Our results revealed that cephalopods with early planktonic developmental modes hatch at smaller sizes and reach broader distributional ranges in comparison with species with large, benthic hatchlings. In addition, for the cephalopod groups with high numbers of species with planktonic hatchlings (squids and octopods), the smaller the hatchling, the broader the latitudinal distributional range of the species. These facts suggest that the developmental mode (planktonic or benthic) along with the hatchling size may influence the distributional range of cephalopods. Furthermore, when the hatchling size index (SHSI) was used for all species with benthic hatchlings, the smaller the hatchlings, the broader the latitudinal distributional range of the species. This also held for the main benthic group, the sepioids. The chances of dispersal by drifting will tend to be greatest in smaller hatchlings. A positive relationship between the presence of a planktonic larval phase and geographic distributional range has been found for prosobranch gastropod species [221], and the present study shows a similar trend for cephalopods.

Lester and Ruttenberg [222] examined the relationship between distributional range and planktonic larval duration of tropical reef fishes and found that this relationship is positively correlated only in the largest ocean basin (the Indo-Pacific). They found that the spatial distribution of habitats and dispersal barriers are of great importance for the dispersal of reef fishes. The authors also noted that the duration of the larval phase is the best quantitative estimate of the dispersal potential of many species. It is conceivable to expect that the smaller the hatchling size, the longer the duration of the planktonic phase. The duration of the planktonic phase in cephalopods has been determined for only 15 species (Table 2), and our results have shown that duration of the planktonic phase increases as hatchling size increases. It is important to stress, however, that this result was strongly influenced by the presence of cold-water octopod species with larger planktonic hatchlings, such as *Enteroctopus dofleini* and *E*. *megalocyathus* (Table 2, Fig 4). Hence, the duration of the planktonic phase in cephalopods seems to be mainly dependent on the species and temperature [7]. Temperature will have a domineering influence, with the potential to increase or decrease the duration of the planktonic phase for a single species. Octopods of the genus *Enteroctopus* have the largest planktonic cephalopod at hatching and the duration of their planktonic phase extends to 4–6 months, influenced by the relatively cold-water temperatures (11°C) where they develop (Table 2). In addition, *E*. *megalocyathus* hatchlings are larger than other octopods at the end of their planktonic phase and settle as juveniles on the bottom, as in *Amphioctopus aegina*, *Octopus joubini*, *O*.*vulgaris* and *Robsonella fontanianus* (see Table 2). It would be highly informative to evaluate the factors regulating the duration of the planktonic phase for many species under controlled conditions. Such information could improve our understanding of the influence of the duration of the planktonic phase on the geographical distribution range of cephalopods.

Cephalopods have a relatively short life cycle, around 1–2 years or less in medium-sized coastal species [4], which implies that the time for dispersal is relatively short in comparison with other long-lived marine groups like fishes. Therefore, the planktonic and juvenile phases last relatively longer in relation to the whole life cycle in cephalopod species and must exert a major influence on their dispersal potential, when compared with other marine animals with longer life cycles. The locomotion capacity of the adults is also closely related to species dispersal, although it has only been studied in a few cephalopod species [223]. In this regard, the short life cycle of coastal cephalopods and their relatively brief adult period suggest again the importance of planktonic and juvenile phases for dispersal.

In marine invertebrates, the offspring size variation can arise from different factors such as maternal size and nutrition, habitat quality and stress [224]. The intraspecific variation in hatchling size has the potential to influence the population dynamics. In cephalopods, this intraspecific variability in hatchling size has been reported for several species (see also Table 1) and attributed mainly to egg incubation temperature [91, 104, 225–227] and the consequent duration of the embryonic phase [228], seasonality [108] as well as maternal influence [229–233]. The effect of temperature on the duration of embryonic development is well known in cephalopods [234]; as temperature increases, the duration of embryonic development decreases, producing smaller hatchlings. On the other hand, at lower temperatures, embryonic development is longer and hatchlings are larger. This temperature effect can occur at seasonal and latitudinal gradients. Moreover, for a single species, larger hatchlings should be expected at higher latitudes [235].

Other factors influencing dispersal that were not considered here include geological history and oceanographic currents. The latter have a domineering effect on dispersal of marine species. For Mediterranean littoral fish species, inshore larvae showed shorter planktonic larval duration than species with offshore larvae. As a result, mean geographic range was smaller for species with inshore larval distribution than for species with offshore larval distribution [236]. These results indicated that planktonic larval duration is certainly not the only factor controlling geographical range, as the main circulation in the inshore-offshore larval habitat as well as the season of planktonic life play important roles in dispersal.

The results of the present study also indicate that hatchling size is related to dispersal potential and display a phenotypic association with the presence of a planktonic phase and developmental mode. Small hatchling size in marine invertebrates is often associated with planktonic developmental mode, high mortality rates, large dispersal potential and likely gene flow. On the other hand, large hatchling size is linked with benthic development and limited dispersal [237]. Genetic data have given general support to the association between developmental mode and intrapopulation variation, with low genetic differentiation being commonly found in planktonic populations, although there are exceptions [238, 239].

Genetic studies in populations of holobenthic octopods showed that individuals are unable to disperse between sites separated by tracts of deep ocean, which apparently present a major physical barrier to dispersal, such as depths > 1000 m for *Pareledone turqueti* [240]. This isolation, mediated by the limited movement of benthic adults, seems to promote the population differentiation pattern in continuous habitats as in the holobenthic *Octopus pallidus* [241]. In contrast, merobenthic octopuses showed less consistent patterns, with interactions of multiple factors, such as oceanic currents, duration of the planktonic phase and fitness with settlement areas influencing species dispersal and connectivity [241]. For *Octopus vulgaris* type II, microsatellite data have revealed significant genetic differentiation in four populations from the SW Atlantic, however, no relationship between geographic distance and genetic differentiation was found [242]. The combination of morphological and microsatellite data, has provide evidences of phylogeographic boundaries for *Loligo reynaudii* in southern Africa [243], despite its narrow distribution range with no obvious physical boundaries. Many topics remain to be investigated, such as the interactions between paralarval swimming behaviour and wind-driven circulation, which may strongly affect dispersal and retention patterns, leading to many possible explanations for genetic and morphologic diversity.

According to Strahmann and Strahmann [244], the recruitment variability inherent to species with small-sized hatchlings and planktonic development seems to be incompatible with short life cycles and low fecundity of small-sized species. Planktonic developmental mode plays both the role in feeding and dispersal. Thus, a greater incidence of planktonic development should be expected in large animals with longer life spans. Interestingly, though, a study that correlated adult size with oocyte size and inferred developmental mode in shallow water benthic octopuses suggested that oocyte size is negatively related to body size and thus, species with larger body sizes tend to have smaller oocytes (and likely planktonic hatchlings) compared to smaller body sized species [171]. It should be noted that octopus species with considerably large body sizes such as *Enteroctopus dofleini and E*. *megalocyathus*, have planktonic hatchlings, in agreement with the suggestions of both Guzik [171] and Strahmann and Strahmann [244]. On the other hand, polar regions seem to have selected for the production of large benthic hatchlings as there is abundant food during the productive summers months minimizing post-settlement competition [245]. This appears to apply particularly to polar and deep-sea octopus species, which have very large hatchlings that are produced over long to exceptionally long incubation periods, as in *Graneledone boreopacifica*, the species with the longest egg brooding period (53 months) ever registered for an animal [246].

The deep-sea and polar octopuses show a clear tendency for large hatchlings. Phylogenetic evidence suggests that polar and deep-sea octopuses, all with benthic hatchlings, have shallow water ancestors with planktonic paralarvae [247, 248]. In the deep-sea, the main incirrate octopods such as *Bathypolypus*, *Benthoctopus* and *Graneledone*, have very large eggs, suggesting benthic hatchlings [249–251]. In this bathyal and abyssal environment, the suborder of the cirrate octopods probably represents an exception to the rule. These typical deep-sea cephalopods with a gelatinous consistency and well-developed fins, spawn very large eggs suggesting direct developing juveniles [252]. However, the cirrate octopod family Cirroteuthidae (*Cirrothauma*, *Cirroteuthis*, *Stauroteuthis*) are essentially pelagic, but live generally close to the sea floor, and are characterized by very large fins and swimming behaviour [253–255]. The Cirroteuthidae morphology suggests a possible planktonic or benthopelagic mode of development for large hatchlings in the deep-sea, because very large fins were observed in advanced cirrate embryos of 9 mm ML [256]. This exception could also be extended to the old cephalopod lineage of the nautiluses, with very large and pelagic hatchlings [44, 45, 257]. Increased dispersal capacity has the potential to impact recruitment variability and thus, may have several consequences for the whole life cycle. It is important to emphasize, however, that dispersal is not only achievable during the larval phase of the life cycle, but that other factors such as adult body size and locomotion capacities also play a role. There may be interesting insights to be gained from exploring the importance of developmental mode and dispersal with gene flow in cephalopod populations.

## Conclusions

Cephalopod species with smaller planktonic hatchlings seem to reach larger distributional extensions in comparison with species with large, benthic hatchlings. This seems evident for squids and octopods, where species with larger hatchlings have geographical distributions of comparatively minor extension. This general tendency was not detected for sepioids, a more homogeneous group composed mainly of species with large and benthic hatchlings. However, when observing the relative size of the hatchlings in comparison with the adults, the smaller the hatchlings, the broader the latitudinal distribution range of sepioids. This was also valid for all species with benthic hatchlings pooled together, thus confirming the influence of hatchling size on dispersal potential. The duration of the planktonic phase is also believed to be an important factor influencing the species geographical distribution. However, this has been determined only for a few cephalopod species to date and future research is needed on this topic.
